# Subspace Learning for Dual High-Order Graph Learning Based on Boolean Weight

**DOI:** 10.3390/e27020107

**Published:** 2025-01-22

**Authors:** Yilong Wei, Jinlin Ma, Ziping Ma, Yulei Huang

**Affiliations:** 1 School of Mathematics and Information Science, North Minzu University, Yinchuan 750021, China; weiyilong0402@163.com (Y.W.); maziping@tom.com (Z.M.); 2 School of Computer Science and Engineering, North Minzu University, Yinchuan 750021, China; 3 School of Mathematics and Statistics, Ningxia University, Yinchuan 750021, China; huangyulei@stu.nxu.edu.cn

**Keywords:** unsupervised feature selection, Subspace learning, dual high-order graph learning, Boolean weight

## Abstract

Subspace learning has achieved promising performance as a key technique for unsupervised feature selection. The strength of subspace learning lies in its ability to identify a representative subspace encompassing a cluster of features that are capable of effectively approximating the space of the original features. Nonetheless, most existing unsupervised feature selection methods based on subspace learning are constrained by two primary challenges. (1) Many methods only predominantly focus on the relationships between samples in the data space but ignore the correlated information between features in the feature space, which is unreliable for exploiting the intrinsic spatial structure. (2) Graph-based methods typically only take account of one-order neighborhood structures, neglecting high-order neighborhood structures inherent in original data, thereby failing to accurately preserve local geometric characteristics of the data. To pursue filling this gap in research, taking dual high-order graph learning into account, we propose a framework called subspace learning for dual high-order graph learning based on Boolean weight (DHBWSL). Firstly, a framework for unsupervised feature selection based on subspace learning is proposed, which is extended by dual-graph regularization to fully investigate geometric structure information on dual spaces. Secondly, the dual high-order graph is designed by embedding Boolean weights to learn a more extensive node from the original space such that the appropriate high-order adjacency matrix can be selected adaptively and flexibly. Experimental results on 12 public datasets demonstrate that the proposed DHBWSL outperforms the nine recent state-of-the-art algorithms.

## 1. Introduction

With the rapid proliferation of high-dimensional data characterized by increasing dimensionality and complexity, dimensionality reduction has emerged as an essential yet challenging aspect of data analysis and interpretation [1]. This necessity is particularly evident in applied fields such as text processing, facial recognition, image recognition, and natural language processing. The primary objective of dimensionality reduction is to eliminate noise and redundant information from high-dimensional datasets while preserving critical information, thereby enhancing the efficiency of these methodologies [2,3]. As two key techniques of dimension reduction, subspace learning and feature selection manage to establish a limited number of discriminative features to effectively minimize noise and redundancy [4,5]. Specifically, subspace learning focuses on mapping high-dimensional data from its original space to a lower-dimensional subspace [6], whereas feature selection concentrates on identifying and retaining the most informative subset of features [7].

Generally, subspace learning methods can be categorized into two main categories: supervised and unsupervised based on the offering of labeled information during the learning process. As one of the classical supervised subspace learning methods, Linear Discriminant Analysis (LDA) [8] employs label information to learn discriminative projection, thereby enhancing inter-class distance and reducing intra-class distance. Classical unsupervised subspace learning involves Principal Component Analysis (PCA) [9], Locally Preserved Projection (LPP) [10], and Locally Linear Embedding (LLE) [11]. PCA identifies a projection that retains the principal variance of the data to capture the overall structure. However, PCA is based on the assumption of linearity, which might result in overlooking significant local structures within the data. In contrast, LPP addresses this limitation by deriving projections based on different geometric structures of the original data. LPP focuses on preserving local information, but it may struggle with global data structures and requires the careful tuning of parameters to achieve optimal performance. Similarly, LLE also aims to preserve the local geometry within each neighborhood, effectively maintaining local relationships among data points. Due to excelling in retaining local features in LLE, it is computationally expensive, leading to it being an arduous task on large datasets. In conclusion, classical subspace learning methods typically fail to fully exploit the local geometric features of data, especially when faced with complex distributions or highly nonlinear datasets. Inevitably, this limitation can lead to information loss and a deterioration in classification performance.

Feature selection is one of the dimension reduction techniques, primarily aimed at identifying the most relevant features while preserving their inherent physical significance. Similarly, due to the offering of labeled information, selection methods can be segmented into supervised and unsupervised approaches. As conventional supervised learning methods, the Fisher Score [12] and Subset-Level Score (SFS) [13] leverage statistical methods to assess the relationship among attributes and classes, effectively identifying important features. The former assesses the discriminative ability of each feature by measuring the ratio of inter-class variance to intra-class variance, whereas the latter utilizes a trace ratio form to calculate the subset-level score to establish a global optimal feature subset. In contrast, in the realm of unsupervised learning, prevalent methods involve the Laplacian Score (LS) [14] and Multi-Cluster Feature Selection (MCFS) [15]. The LS constructs a graph-based representation by embedding the manifold regularization constraints to measure the inherent relevance between features, whereas MCFS retains the multi-cluster structure of the data by fully taking account of the potential correlations among diverse features. It is worth noting that the limitations of the above feature selection methods stem from their reliance on predefined statistical metrics, which might result in insufficiently capturing the complex relationships inherent in the data. From another view, this dependence tends to lead to the omission or suboptimal selection of discriminative features or high sensitivity to noise that will arise within complex data structures [16,17].

To tackle the challenges of subspace learning and feature selection, a fused framework has recently been established by integrating the strengths and advantages of these two kinds of approaches [18,19]. In detail, the essence of these methods is to convert unsupervised feature selection into a matrix factorization problem from the perspective of subspace learning, thereby effectively distinguishing the most critical feature subspaces consistent wtih the original space [20,21,22]. Inevitably, in unsupervised feature selection methods based on subspace learning methods, focusing more on capturing the global structure rather than the local structure of the data hinders sample generalization [23]. To alleviate this, recent research has conducted subspace learning with discriminative information by generating pseudo-label information, simultaneously embedding various orthogonal constraint strategies to guide feature selection, thereby highlighting more local information [24,25]. Alternatively, one of the fundamental concepts that excels in uncovering local structures is “manifold learning” which underscores the similarity between two subspaces [26]. Guided by geometric structure information on the feature manifold, the process of learning the feature selection matrix and coefficient matrix will be accelerated, and thus a more accurate manifold structure will be extracted [27]. Similarly, incorporating both local manifold structure information and local discriminative information could yield a more effective feature selection process [28]. Notably, the above-mentioned methods succeed in constructing a fixed graph structure throughout the optimization process but lack the flexible capability to accommodate potential variations and complexities of diverse datasets. For this reason, an adaptive similarity matrix is constructed to learn higher-quality graph structures, thereby preserving more robust global reconstruction information and local geometric structures and offering a flexible learning mechanism [29]. Building on this foundation, the concept of maximizing the inter-class scatter matrix is accomplished by incorporating the trace ratio approach and adaptive graph learning methods, guiding the feature selection process to capture richer and more detailed manifold information [30].

In summary, the aforementioned methods build a bridge between feature selection and other essential concepts such as subspace learning and manifold learning, but they suffer from two notable limitations: (1) The correlated information between both features and samples is not taken into account simultaneously. (2) Compared with the basic facts in real data, simple one-order adjacency matrices always lack certain critical connections to fully exploit the complex structure inherent in the data.

To solve these problems, an unsupervised feature selection method named DHBWSL is proposed in this paper. The main contributions to this are summarized as follows:

- A novel unsupervised feature selection method is proposed that captures structural information more comprehensively by considering hidden high-order similarities in both data space and feature space. A new discovery of DHBWSL is the adaptive learning of dual high-order adjacency matrices, the dynamic adjustment of the graph structure, and the selection of discriminative features within a unified framework.
- We design an adaptive dual high-order graph learning mechanism by associating Laplacian rank constraints with Boolean variables to adaptively learn adjacency matrices with consensus structures from suitable high-order adjacency matrices, thereby enhancing the quality of the graph structure.
- Extensive experiments conducted on 12 public datasets demonstrate that DHBWSL outperforms the performance of various leading unsupervised feature selection models.

The rest of the paper is organized as follows: Section 2 briefly describes the related work. In Section 3, our proposed method is presented, including the objective function, the alternating iteration scheme for solving the optimization problem, and the computational complexity and convergence analysis. Extensive experiments are conducted to demonstrate the effectiveness and superiority of DHBWSL compared to the state-of-the-art methods in Section 4. Finally, Section 5 concludes the paper.

## 2. Related Work

In this section, we provide an overview of the mathematical symbols used in this paper and primarily review some of the work closely related to our algorithm.

### 2.1. Related Notations

Let $X^{T}$ and $Tr\left(X\right)$ specify the transpose and trace of the matrix X, and let l represent the number of chosen features (l ≤ d). The norm of any matrix X is defined as: ${\left||X|\right|}_{r,s}={\left(\sum_{i=1}^{d}{\left(\sum_{j=1}^{n}{\left|X_{ij}\right|}^{r}\right)}^{s/r}\right)}^{1/s}$. Based on the previous definition, the norm is known as the Frobenius norm or the *ℓ*_2_-norm for r=s= 2. When r=2 and s=1, the norm is known as the *ℓ*_2,1_-norm. For clarity, we summarize the basic notations employed in this research in Table 1.

### 2.2. MFFS

Motivated by the principles of subspace learning, MFFS is formulated as a feature selection method using a projection matrix [20]. It is assumed that all features reside within a linear manifold within the true feature space, allowing feature selection to be achieved by approximating the high-dimensional original space through a minimal set of low-dimensional subspaces.

The goal of subspace learning is to depict the distance between the raw data matrix *X* and the feature subset *X_I_*:

(1) $${\left\|X-X_{I}V\right\|}_{F}^{2},\;s.t.\;\left|I\right|=l,$$

where *I* is the index set of the selected features, and |*I*| is the number of elements in it, *V* is the coefficient matrix of the initial feature space, and *l* represents the number of selected features.

From the viewpoint of matrix factorization, the feature selection problem is expressed as follows:

(2) $${\left\|X-XZV\right\|}_{F}^{2},\;s.t.Z\geq 0,\;V\geq 0,Z^{T}Z=I_{l},$$

where *Z* is the feature selection matrix and *I_l_* is the *l* × *l* identity matrix.

### 2.3. CLR

To accomplish this concept of structured adjacency matrix learning derived from graph theory, the Constrained Laplacian Rank (CLR) intended to dynamically refine the graph structure *S*, learning a new data graph *S* from a given data graph *A* to guarantee that the resulting graph *S* contains exactly *k* connected components, where *k* corresponds to the number of clusters.

For a non-negative affinity matrix *A*, the Laplacian matrix $L_{A}=D_{A}-\frac{\left(A^{T}+A\right)}{2}$, where *D_A_* is the diagonal matrix whose ith element is $\sum_{j}\frac{\left(a_{ij}+a_{ji}\right)}{2}$, has the following crucial feature [31]:

**Theorem** **1.** *The multiplicity k of the eigenvalue zero of the Laplacian matrix L_A_ equals the number of linked components in the graph associated with A.*

According to **Theorem 1**, the graph is perfect if $rank(L_{S})=n-k$. To avoid the situation in which some rows of *S* are entirely zero, we further limit *S* such that the total of each row is one. The CLR optimizes the following issue:

(3) $${\left||S-A|\right|}_{F}^{2},\;s.t.\sum_{j}s_{ij}=1,\;s_{ij}\geq 0,rank(L_{S})=n-k,$$

addressing this optimization challenge is complex due to the fact that *L_S_* is defined as $L_{S}=D_{S}-\frac{\left(S^{T}+S\right)}{2}$, where *D_S_* depends on *S*, and the constraint $rank(L_{S})=n-k$ is a complicated nonlinear constraint. The particular solution’s steps will be detailed in depth in the next section.

## 3. Methodology

In this section, we propose a novel unsupervised feature selection model called DHBWSL. The concept of DHBWSL is illustrated by the following dual high-order graph learning as well as its most important properties, such as the optimization process and convergence analysis.

### 3.1. Dual High-Order Graph Learning

In general, similar graphs are limited to capturing pairwise relationships between data points and can only reflect one-order neighbor relationships. However, these pairwise relationships are highly sensitive to changes in the parameters of the nearest neighbor; in other words, even small adjustments can significantly alter the matrix structure, thereby diminishing clustering performance. To address these challenges, we propose the new concept “neighbors of neighbors are also neighbors” that is capable of exploiting more node information to construct a high-order adjacency matrix, which contributes to improving the robustness of nearest-neighbor parameters and better capturing the structural characteristics of the data.

Specifically, based on the definition of one-order adjacency matrix $S_{1\;}$ ∈ R, we introduce the concept of high-order adjacency matrix $S_{n}=S_{n-1}\times S$ ∈ $R^{n}(n=2,\cdots ,O)$, where *O* denotes the max order of S. Considering that there may be multiple path interactions between data points, a high-order adjacency matrix can enrich the diversity of local structural information and highlight more representative and distinguished features. For complementing intra-class and inter-class information, we are inspired to learn a uniform adjacency matrix by applying a multi-order adjacency matrix from different paths.

(4) $$\sum_{i=1}^{O}{\left||W-S_{i}|\right|}_{F}^{2},\;s.t.\;W1=1,W\in R^{+},rank(L_{W})=n-K,$$

where *L_W_* is the Laplacian matrix.

Although high-order adjacency matrices are more robust than one-order adjacency matrices in dealing with the sensitivity of the nearest neighbor parameter *k*, it is difficult to find the balance to determine the most suitable of the number of high-order adjacency matrices. The main reason may lie in that the lower order readily results in the failure of the adjacency matrix to be appropriately consistent with the intricate relationships in the data, potentially accompanying the loss of vital information, whereas a high-order might cause negative effects such as superfluous duplicate information, increasing the model complexity, and overtraining.

To address the above issues, we set the order *O* to a considerably elevated value to contain a wider number of alternatives. Then, we choose *M* relevant subsets of $\varepsilon_{O}^{M}$. To find the proper order, we use an adaptive order adjacency-learning approach that minimizes the *M* residuals of $\left\{{\left||W-S_{i}|\right|}_{F}^{2}|i\in \{1,2,\cdots ,O\}\right\}$:

(5) $$\sum_{i=1}^{O}p_{i}{\left||W-S_{i}|\right|}_{F}^{2},\;s.t.\;W1=1,W\in R^{+},rank\left(L_{W}\right)=n-K,p^{T}1=M,p\in {\left\{0,1\right\}}^{O},$$

where $p_{i}$ is a Boolean variable that specifies whether to use the *i*-th order adjacency matrix $S_{i}$.

Given that the rank limitation on the Laplace matrix *L* is a strong constraint, solving Equation (5) directly appears to be quite difficult. To address this issue, we relax the rank constraint on the Laplacian matrix *L* by decreasing its rank requirement. In the data space, we suppose that $\sigma_{i}\left(L^{V}\right)$ signifies the i-th lowest eigenvalue of the Laplace matrix $L^{V}$. For any *i*, there is $\sigma_{i}\left(L^{V}\right)$ ≥ 0. When *β* is big enough, the best solution $W^{V}$ for solving the problem (5) equals the second term $\sum_{i=1}^{K}\sigma_{i}\left(L^{V}\right)=0$. Equation (5) transforms into:

(6) $$\sum_{i=1}^{O}p_{i}{\left||W^{V}-S_{i}^{V}|\right|}_{F}^{2}+\beta \sum_{i=1}^{K}\sigma_{i}\left(L^{V}\right),\;s.t.W^{V}1=1,W^{V}\in R^{+},p^{T}1=M,p\in {\left\{0,1\right\}}^{O},$$

where *β* is the regularization parameter and *W^V^* is the structure matrix of the data space.

According to the Ky Fan Theorem (Fan 1949) [32], for every Laplacian matrix *L*, the sum of its first *K* minimum eigenvalues may be calculated by solving the following minimization problem as follows:

(7) $$\sum_{i=1}^{K}\sigma_{i}\left(L^{V}\right)=Tr\left(VL^{V}V^{T}\right),$$

therefore, the issue (6) is also equal to:

(8) $$\sum_{i=1}^{O}p_{i}{\left||W^{V}-S_{i}^{V}|\right|}_{F}^{2}+\beta Tr\left(VL^{V}V^{T}\right),\;s.t.W^{V}1=1,W^{V}\in R^{+},V\geq 0,p^{T}1=M,p\in {\left\{0,1\right\}}^{O},$$

where *β* is the equilibrium parameter and *V* is the coefficient matrix.

### 3.2. The Proposed Feature Selection Method

This section describes the objective function of DHBWSL as follows:

(9) $$O={\left\|X^{T}-X^{T}HV\right\|}_{F}^{2}+\sum_{i=1}^{O}p_{i}^{V}{\left||W^{V}-S_{i}^{V}|\right|}_{F}^{2}+\beta Tr(VL^{V}V^{T})+\sum_{i=1}^{O}p_{i}^{U}{\left||W^{U}-S_{i}^{U}|\right|}_{F}^{2}+\gamma Tr(H^{T}XL^{U}X^{T}H)+\lambda {\left\|H\right\|}_{2,1},\;s.t.H\geq 0,\;V\geq 0,H^{T}H=I_{l},W^{V}1=1,W^{V}\in R^{+},W^{U}1=1,W^{U}\in R^{+},{\left(p_{i}^{U}\right)}^{T}1=M,p_{i}^{U}\in {\left\{0,1\right\}}^{O},{\left(p_{i}^{V}\right)}^{T}1=M,p_{i}^{V}\in {\left\{0,1\right\}}^{O}.$$

In Equation (9) above, the first term integrates subspace learning to effectively reduce dimensionality and filter out noise. It projects the data into a lower-dimensional space, thereby capturing fundamental feature relationships and enhancing the model’s robustness and efficiency. The second and third terms employ adaptive mechanisms to account for high-order feature interactions, transcending simple pairwise relationships to consider complex synergies among multiple features. The fourth and fifth terms related to higher-order data point distributions consider the high-order relationships between data points, thereby more accurately preserving the original data’s local structure. Lastly, the sixth term enforces sparsity, promoting the selection of only the most relevant features. This results in a compact and interpretable model, enabling a more precise identification of feature subsets.

**Figure 1:**
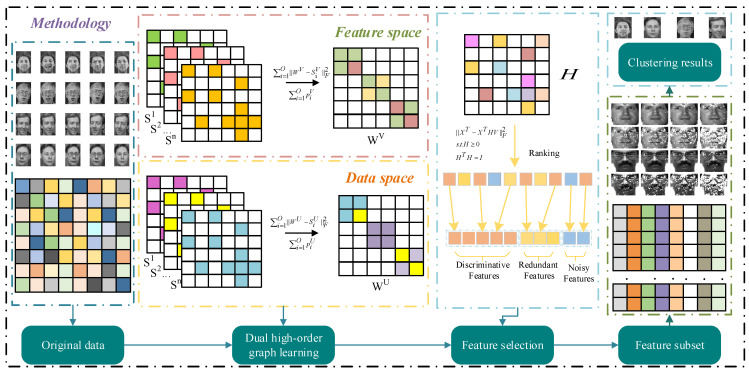
The framework of the DHBWSL method.

The regularization parameters *β* and *γ* balance the smoothness of the data and feature space, whereas *λ* is a sparsity constraint parameter used to modify *H* ∈ *R^d^*^×*l*^. To simplify the formula, define *U* ∈ *R^n^*^×*l*^ as the product of variables *X^T^* and *H*, i.e., *U* = *X^T^H*. The feature transformation matrix *H* optimizes the score for each feature ‖*h_i_*‖_2_ to reflect its importance. High scores indicate more significant aspects. To generate the new data matrix *X_new_*, we sorted the scores in decreasing order and chose the top *l* features from the original dataset of *d* features. The overall framework of DHBWSL is shown in Figure 1.

### 3.3. Comparison of Unsupervised Feature Selection Based on Subspace Learning

To evaluate the clustering performance of DHBWSL, the following representative subspace learning-based unsupervised feature selection methods are summarized as Table 2 for the comparison task.

### 3.4. Optimization

In this section, a new efficient approach is introduced for solving with the primary goal of minimizing the objective function (9) [38,39]. To establish restrictions on *H_ij_* ≥ 0 and *V_ij_* ≥ 0, two Lagrange multipliers, *ϕ_ij_* and *ψ_ij_*, are introduced. Hence, the Lagrange function of Equation (9) is as follows:

(10) $$L=Tr((X^{T}-X^{T}HV){\left(X^{T}-X^{T}HV\right)}^{T})+\sum_{i=1}^{O}p_{i}^{V}{\left||W^{V}-S_{i}^{V}|\right|}_{F}^{2}+\beta Tr(VL^{V}V^{T})+\sum_{i=1}^{O}p_{i}^{U}{\left||W^{U}-S_{i}^{U}|\right|}_{F}^{2}+\gamma Tr(H^{T}XL^{U}X^{T}H)+\lambda Tr(H^{T}QH)+\frac{\sigma }{2}Tr(\left(H^{T}H-I_{l}\right){\left(H^{T}H-I_{l}\right)}^{T})+Tr\left(\phi H\right)+Tr\left(\psi V\right),$$

where *σ* is an orthogonal constraint parameter, $Q=[q_{ij}]$∈$R^{d\times d}$ is a diagonal matrix with the *i*-th diagonal member $q_{ii}$ represented as:

(11) $$q_{ii}=\frac{1}{{2||h_{i}||}_{2}},$$

to solve Equation (11), we insert a minor constant *ε* to prevent overflow:

(12) $$q_{ii}=\frac{1}{{2max(||h_{i}||}_{2},\varepsilon )}.$$

#### 3.4.1. Update V and H

(1) Fix *V* and update *H*:

Set the partial derivative of *L*(*H*,*V*) with respect to *H* to zero,

(13) $$\frac{\partial L}{\partial H}=-2XX^{T}V^{T}+2XX^{T}HVV^{T}+2\gamma XL^{U}X^{T}H+2\lambda QH+2\delta {HH}^{T}H-2\delta H+\phi ,$$

applying the KKT requirements [40], $\phi_{ij}H_{ij}=0$, we obtain:

(14) $${\left[-2XX^{T}V^{T}+2XX^{T}HVV^{T}+2\gamma XL^{U}X^{T}H+2\lambda QH+2\delta {HH}^{T}H-2\delta H\right]}_{ij}H_{ij}=0,$$

using *L^U^* = *D^U^* – *W^U^*, we obtain the iterative updating procedure for H:

(15) $$H_{ij}\leftarrow H_{ij}\frac{{\left[XX^{T}V^{T}+\gamma {XW}^{U}X^{T}+\delta H\right]}_{ij}}{{\left[XX^{T}HVV^{T}+\gamma XD^{U}X^{T}H+\lambda QH+\delta {HH}^{T}H\right]}_{ij}}.$$

(2) Fix *H* and update *V*:

Taking the partial derivative of Equation (10) with regard to *V* yields:

(16) $$\frac{\partial L}{\partial V}=-2{H^{T}XX}^{T}+2H^{T}XX^{T}HV+2\beta VL^{V}+\psi ,$$

using the KKT conditions [40], $\psi_{ij}V_{ij}=0,$ we obtain:

(17) $${\left[-2{H^{T}XX}^{T}+2H^{T}XX^{T}HV+2\beta VL^{V}\right]}_{ij}V_{ij}=0,$$

using *L^V^* = *D^V^* − *W^V^*, we obtain the iterative update procedure for *V*:

(18) $$V_{ij}\leftarrow V_{ij}\frac{{\left[{H^{T}XX}^{T}+\beta {VW}^{V}\right]}_{ij}}{{\left[H^{T}XX^{T}HV+\beta VD^{V}\right]}_{ij}}.$$

#### 3.4.2. Update p^V^ and W^V^

We only consider the variables *p^V^* and *W^V^* due to the symmetry of the data and feature spaces. The same applies to *p^U^* and *W^U^*.

(1) Fix *W^V^* and update *p^V^*:

(19) $$\sum_{i=1}^{O}p_{i}^{V}{\left||W^{V}-S_{i}^{V}|\right|}_{F}^{2},\;s.t.{\left(p_{i}^{V}\right)}^{T}1=M,p_{i}^{V}\in {\left\{0,1\right\}}^{O},$$

which has a closed-form solution as follows:

(20) $$p_{i}^{V}=\left\{\begin{array}{cc} 1, & if\;{\left||W^{V}-S_{i}^{V}|\right|}_{F}^{2}\leq {\left||W^{V}-S_{M}^{V}|\right|}_{F}^{2}\\ 0, & Otherwise \end{array}\right.,$$

where ${\left||W^{V}-S_{M}^{V}|\right|}_{F}^{2}$ is the *M*-th smallest value from the set $\left\{{\left||W^{V}-S_{i}^{V}|\right|}_{F}^{2}|i\in \{1,2,\cdots ,O\}\right\}$.

After removing the items that are not related to *p^U^*, we may update *p^U^* by solving the following issue using the preceding method:

(21) $$\sum_{i=1}^{O}p_{i}^{U}{\left||W^{U}-S_{i}^{U}|\right|}_{F}^{2},\;s.t.{\left(p_{i}^{U}\right)}^{T}1=M,p_{i}^{U}\in {\left\{0,1\right\}}^{O},$$

(2) Fix *p^V^* and update *W^V^*:

(22) $$\sum_{i=1}^{O}p_{i}^{V}{\left||W^{V}-S_{i}^{V}|\right|}_{F}^{2},\;s.t.W^{V}1=1,W^{V}\in R^{+},rank\left(L^{V}\right)=d-K.$$

according to:

(23) $$\sum_{i=1}^{O}p_{i}{\left\|W^{V}\right\|}_{F}^{2}-2Tr({\left(W^{V}\right)}^{T}\sum_{i=1}^{O}p_{i}S_{i}^{V})+\sum_{i=1}^{O}p_{i}{\left||S_{i}^{V}|\right|}_{F}^{2}⟹{\left\|W^{V}\right\|}_{F}^{2}-Tr({\left(W^{V}\right)}^{T}\frac{2\sum_{i=1}^{O}p_{i}S_{i}^{V}}{\sum_{i=1}^{O}p_{i}}).$$

The concerns involved can be characterized as the intrinsic challenges of the CLR technique, which are discussed below:

(24) $${\left||W^{V}-\frac{\sum_{i=1}^{O}p_{i}S_{i}^{V}}{\sum_{i=1}^{O}p_{i}}|\right|}_{F}^{2},\;s.t.W^{V}1=1,W^{V}\in R^{+},rank\left(L^{V}\right)=d-K.$$

In another form, when $A^{V}=\frac{\sum_{i=1}^{O}p_{i}S_{i}^{V}}{\sum_{i=1}^{O}p_{i}}$, we can obtain:

(25) $${\left\|W^{V}-A^{V}\right\|}_{F}^{2},\;s.t.W^{V}1=1,W^{V}\in R^{+},rank\left(L^{V}\right)=d-K.$$

**Theorem 1**, described in Section 3.1 [32], transforms the constraint $rank\left(L^{V}\right)=d-K$ into $Tr\left(VL^{V}V^{T}\right)$:

(26) $${\left\|W^{V}-A^{V}\right\|}_{F}^{2}+\beta Tr\left(VL^{V}V^{T}\right),\;s.t.W^{V}1=1,W^{V}\in R^{+},V\geq 0,$$

where *β* is the graph regularization parameter, which converts to vector form when *V* is fixed:

(27) $$\sum_{i,j=1}^{d}{\left(w_{ij}^{V}-a_{ij}^{V}\right)}^{2}+\beta \sum_{i,j=1}^{d}{\left||v_{i}-v_{j}|\right|}_{2}^{2}w_{ij}^{V},\;s.t.\sum_{j=1}^{d}w_{ij}^{V}=1,0\leq w_{ij}^{V}\leq 1.$$

For distinct *i*, issue (27) is independent; hence, we can solve the following problems separately:

(28) $$\sum_{j=1}^{d}{\left(w_{ij}^{V}-a_{ij}^{V}\right)}^{2}+\beta \sum_{j=1}^{d}{\left||v_{i}-v_{j}|\right|}_{2}^{2}w_{ij}^{V},\;s.t.\sum_{j=1}^{d}w_{ij}^{V}=1,0\leq w_{ij}^{V}\leq 1.$$

Using $m_{ij}^{V}={\left||v_{i}-v_{j}|\right|}_{2}^{2}$, and $m_{i}^{V}$ as a vector with *j*-th element equal to $m_{ij}^{V}$ (and similarly for $a_{ij}^{V}$), issue (28) may be written in vector form as follows:

(29) $$\sum_{j=1}^{d}{\left||w_{j}^{V}-(a_{j}^{V}-\frac{\beta }{2}m_{j}^{V})|\right|}_{2}^{2},\;s.t.{\left(w_{i}^{V}\right)}^{T}1=1,0\leq w_{ij}^{V}\leq 1.$$

This issue may be solved using either a closed-form solution, as stated in Equation (29), or an efficient iterative technique. When $h_{j}^{V}=-(a_{j}^{V}-\frac{\beta }{2}m_{j}^{V})$, we have:

(30) $$\sum_{j=1}^{d}{\left||w_{j}^{V}+h_{j}^{V}|\right|}_{2}^{2},\;s.t.{\left(w_{i}^{V}\right)}^{T}1=1,0\leq w_{ij}^{V}\leq 1,$$

where *τ* and $g_{i\;}$≥ 0 are the Lagrangian multipliers. Without the loss of generality, we set *k* to 1/2. Using the KKT criteria [40], the following equation is derived:

(31) $$\left\{\begin{array}{c} \forall j,{\left(w_{ij}^{V}\right)}^{*}+h_{ij}^{*}-\tau^{*}-g_{ij}^{*}=0\\ \forall j,{\left(w_{ij}^{V}\right)}^{*}g_{ij}^{*}=0\\ \forall j,{\left(w_{ij}^{V}\right)}^{*}\geq 0\\ \forall j,g_{ij}^{*}\geq 0 \end{array}\right.,$$

where ${\left(w_{ij}^{V}\right)}^{*}$ represents the j-th element of ${\left(w_{i}^{V}\right)}^{*}$. Using the restriction ${\left(w_{i}^{V}\right)}^{T}1=1$, we obtain:

(32) $$\tau^{*}=\frac{1+1^{T}h_{i}-1^{T}g_{i}^{*}}{d},$$

combining (32) with the first term of (31), we obtain:

(33) $${\left(w_{ij}^{V}\right)}^{*}=\frac{1}{d}\left(1-1^{T}g_{i}^{*}\right)1-(h_{i}-\frac{1}{d}1^{T}h_{i}1)+g_{i}^{*},$$

and:

(34) $${\left(w_{ij}^{V}\right)}^{*}=\frac{1}{d}-\frac{1^{T}g_{i}^{*}}{d}-h_{ij}+\frac{1^{T}h_{i}}{d}+g_{ij}^{*}.$$

To prevent misunderstandings, remember that $1^{T}h_{i}1=(1^{T}h_{i})1$, where $1^{T}h_{i}$ represents a constant. Furthermore, we designate $\bar{g_{i}^{*}}=(1^{T}g_{i}^{*}/d)$, $e_{ij}=(1/d)-(h_{ij})1^{T}h_{i}$, and ${\left(w_{ij}^{V}\right)}^{*}$ as follows:

(35) $${\left(w_{ij}^{V}\right)}^{*}=e_{ij}+g_{ij}^{*}-\bar{g_{i}^{*}}.$$

Given the third and fourth terms in Equation (31), Equation (35) may be substituted:

(36) $${\left(w_{ij}^{V}\right)}^{*}={\left(e_{ij}-\bar{g_{i}^{*}}\right)}_{+}.$$

Identifying the ideal $\bar{g_{i}^{*}}$ yields the optimal solution ${\left(w_{i}^{V}\right)}^{*}$. Equation (36) states that $\bar{g_{i}^{*}}={\left(w_{ij}^{V}\right)}^{*}$+$\bar{g_{i}^{*}}$-$e_{ij}$ and $g_{ij}^{*}={\left(\bar{g_{i}^{*}}-e_{ij}\right)}_{+}$. Averaging the variable $g_{ij}^{*}$ yields:

(37) $$\bar{g_{i}^{*}}=\frac{1}{d}\sum_{j=1}^{d}{\left(\bar{g_{i}^{*}}-e_{ij}\right)}_{+}.$$

The best value of $\bar{g_{i}^{*}}$ may be determined using the Newton approach and a cost function. The cost function is defined as the following:

(38) $$\Phi \left(\bar{g_{i}}\right)=\frac{1}{d}\sum_{j=1}^{d}{\left(\bar{g_{i}^{*}}-e_{ij}\right)}_{+}-\bar{g_{i}}.$$

If the cost function $\Phi \left(\bar{g_{i}}\right)=0$, we have the optimum $\bar{g_{i}^{*}}$. The update rules for the *t* + 1-th iteration are as follows:

(39) $${\bar{g_{i}}}^{t+1}={\bar{g_{i}}}^{t}-\Phi ({\bar{g_{i}}}^{t}){\left[\frac{\partial \Phi \left({\bar{g_{i}}}^{t}\right)}{\partial {\bar{g_{i}}}^{t}}\right]}^{-1},$$

the following features contribute to the effectiveness of the Newton method: $\bar{g_{i}}$≥0, $\partial \Phi \left({\bar{g_{i}}}^{t}\right)/\partial {\bar{g_{i}}}^{t}$ and $\Phi \left(\bar{g_{i}}\right)$ being a piecewise linear convex function.

After eliminating items unrelated to *W^U^*, we may apply the previous strategy to address the following problem:

(40) $$\sum_{i=1}^{O}p_{i}^{U}{\left||W^{U}-S_{i}^{U}|\right|}_{F}^{2},\;s.t.W^{U}1=1,W^{U}\in R^{+},rank\left(L^{U}\right)=n-K.$$

### 3.5. Convergence Analysis

This section provides a convergence analysis of DHBWSL and theoretically proves that the objective function in Equation (9) exhibits monotonically decreasing properties under the iterative update rules (15).

**Definition** **1.** *According to Lee and Seung [41], if the following criteria are met:*

(41) $$M(x,\;x^{\prime })\geq G(x),\;M(x,\;x)=G(x),$$

where $M(x,\;x^{\prime })$ is an auxiliary function for *G*(*x*). Then, *G*(*x*) is monotonically decreasing, and the modified formula is as follows:

(42) $$x^{t+1}arg\underset{x}{\min}M(x,x^{t}).$$

**Proof of Definition 1.**  $G(x^{t+1})$ ≤ $M(x^{t+1},x^{t})$ ≤ $M(x^{t},x^{t})=G(x^{t})$ and *G*(*x*) is convergent.

To construct the following function, we selectively keep the terms involving *H* from Equation (9).

(43) $$G\left(H\right)={\left\|X^{T}-X^{T}HV\right\|}_{F}^{2}+\gamma Tr\left(H^{T}XL^{U}X^{T}H\right)+\lambda {\left\|H\right\|}_{2,1}+\frac{\sigma }{2}{\left\|H^{T}H-I_{l}\right\|}_{F}^{2}=Tr(\left(X^{T}-X^{T}HV\right){\left(X^{T}-X^{T}HV\right)}^{T})+\gamma Tr\left(H^{T}XL^{U}X^{T}H\right)+\lambda Tr\left(H^{T}QH\right)+\frac{\sigma }{2}Tr(\left(H^{T}H-I_{l}\right){\left(H^{T}H-I_{l}\right)}^{T}),$$

By calculating the first- and second-order partial derivatives of *G*(*H*) with respect to *H*, we obtain:

(44) $$G_{ij}^{\prime }={\left[\frac{\partial G}{\partial H}\right]}_{ij}={\left[-2XX^{T}V^{T}+2XX^{T}HVV^{T}+2\gamma XL^{U}X^{T}H+2\lambda QH+2\delta {HH}^{T}H-2\delta H\right]}_{ij},$$

(45) $$G_{ij}^{\prime \prime }=2{\left[XX^{T}\right]}_{ii}{\left[VV^{T}\right]}_{jj}+{\left[2\gamma XL^{U}X^{T}+2\lambda Q+2\delta {HH}^{T}-2\delta I\right]}_{ii},$$

□

**Lemma** **1.** *Define the auxiliary functions of G_ij_ as:*

(46) $$M(H_{ij},\;H_{ij}^{t})=G_{ij}(H_{ij}^{t})+G_{ij}^{\prime }{(H}_{ij}^{t})(H_{ij}-H_{ij}^{t})+\frac{{\left[XX^{T}HVV^{T}+\gamma XD^{U}X^{T}H+\lambda QH+\delta {HH}^{T}H\right]}_{ij}}{H_{ij}^{t}}{\left(H_{ij}-H_{ij}^{t}\right)}^{2},$$

Taylor expansion of $G_{ij}(H_{ij})$ considered:

(47) $$G_{ij}(H_{ij})=G_{ij}(H_{ij}^{t})+G_{ij}^{\prime }{(H}_{ij}^{t})(H_{ij}-H_{ij}^{t})+{\left[XX^{T}VV^{T}+\gamma XL^{U}X^{T}+\lambda Q+\delta HH^{T}-\delta I\right]}_{ij}{\left(H_{ij}-H_{ij}^{t}\right)}^{2}.$$

By establishing Equations (46) and (47), we obtain $M(H_{ij},\;H_{ij}^{t})$ ≥ $G_{ij}(H_{ij})$ inequality. This is comparable to:

(48) $$\frac{{\left[XX^{T}HVV^{T}+\gamma XD^{U}X^{T}H+\lambda QH+\delta {HH}^{T}H\right]}_{ij}}{H_{ij}^{t}}\geq {\left[XX^{T}VV^{T}+\gamma XL^{U}X^{T}+\lambda Q+\delta HH^{T}-\delta I\right]}_{ij}.$$

There are obviously:

(49) $${\left[XX^{T}HVV^{T}+\lambda QH+\delta {HH}^{T}H\right]}_{ij}=\sum_{k=1}^{d}{\left[XX^{T}\right]}_{ik}H_{kj}^{t}{\left[VV^{T}\right]}_{jj}+{\left[\lambda Q+\delta {HH}^{T}\right]}_{ik}H_{kj}^{t}\geq {\left[XX^{T}\right]}_{ii}H_{ij}^{t}{\left[VV^{T}\right]}_{jj}+{\left[\lambda Q+\delta HH^{T}\right]}_{ii}H_{ij}^{t},$$

and:

(50) $$\gamma {\left[XD^{U}X^{T}H\right]}_{ij}=\gamma \sum_{k=1}^{d}{\left[XD^{U}X^{T}\right]}_{ik}H_{kj}^{t}\geq \gamma {\left[XD^{U}X^{T}\right]}_{ii}H_{ij}^{t}\geq \gamma {\left[X\left(D^{U}-W^{U}\right)X^{T}\right]}_{ii}H_{ij}^{t}=\gamma {\left[XL^{U}X^{T}\right]}_{ii}H_{ij}^{t},$$

Equations (49) and (50) hold, the inequality $M(H_{ij},\;H_{ij}^{t})$ ≥ $G_{ij}(H_{ij})$ holds, and the equation $M(H_{ij},\;H_{ij}^{t})=G_{ij}(H_{ij})$ holds when $H_{ij}=H_{ij}^{t}$.

The monotonically declining update rule is then shown to apply to variable H:

**Proof of Lemma 1.**  By substituting the auxiliary function $M(H_{ij},\;H_{ij}^{t})$ into Equation (42), we derive the following update rule:

(51) $$H_{ij}^{t+1}=H_{ij}^{t}-H_{ij}^{t}\frac{G_{ij}^{\prime }(H_{ij}^{t})}{{2[XX^{T}HVV^{T}+\gamma XD^{U}X^{T}H+\lambda QH+\delta {HH}^{T}H]}_{ij}}=H_{ij}^{t}\frac{{\left[XX^{T}V^{T}+\gamma {XW}^{U}X^{T}+\delta H\right]}_{ij}}{{\left[XX^{T}HVV^{T}+\gamma XD^{U}X^{T}H+\lambda QH+\delta {HH}^{T}H\right]}_{ij}}.$$

Based on the above derivation, we can conclude that under the update rule for *H*, the objective function is monotonically decreasing. Similarly, the update rules for other variables also ensure that the objective function value decreases monotonically, thereby guaranteeing their convergence. □

Based on the analysis presented above, Algorithm 1 explains the DHBWSL process.
**Algorithm 1** The procedure of DHBWSL.**Input:** Data matrix *X* ∈ *R^d^*^×*n*^; Parameter *β*, *γ*, *λ*, *σ* and *k*; The number of selected features *l*; The maximum number of iterations *NIter*. **Initialization:** The iteration time *t* = 0; *H* = *rand*(*d*,*l*), *V* = *rand*(*l*,*d*), *I_l_* = *eye*(*l*), Construct the attribute score matrix *Q*;  **Repeat:**  1. Update the feature selection matrix *H* with Equation (15).  2. Update coefficient matrix *V* with Equation (18).  3. Update $p_{i}^{V}$ by solving subproblem (20).  4. Update $p_{i}^{u}$ by solving subproblem (21).  5. Update *W^V^* by solving subproblem (39).  6. Update *W^u^* by solving subproblem (40).  **Until** Convergence **Output:** Index of selected features; New data matrix *X_new_* ∈ *R^l^*^×*n*^. **Feature selection:** The score of *d* features is calculated according to ‖*h_i_*‖^2^, and the first *l* feature with the highest score is selected.

## 4. Experiments

In this section, we evaluate the performance of DHBWSL by comparing with related methods on a public dataset, and provide the parameter sensitivity analysis and convergence analysis. All experiments are implemented in MATLAB 2021b, and run on a Windows machine with 3.20 GHz, i7-75800H, 16 GB of main memory.

### 4.1. Datasets

To evaluate the effectiveness of DHBWSL in terms of clustering performance, 12 public datasets are used in the following experiments, including JAFFE, COIL20, ORL, lung, Isolet, EYale B, TOX_171, GLIOMA, RELATHE, ALLAML, orlraws10P, and COIL100 [33,34,35,36,37,42,43], downloaded at https://jundongl.github.io/scikit-feature/datasets.html, accessed on 3 August 2023, and https://www.face-rec.org/databases/, accessed on 3 August 2023, and Table 3 illustrates the details of these datasets.

### 4.2. Comparison Methods

Since DHBWSL is a UFS method, the comparison experiments are conducted under unsupervised conditions. Nine state-of-the-art unsupervised feature selection algorithms are employed to highlight the superiority of the proposed method. We selected the Baseline method as it offers a fundamental reference point, emphasizing the importance of feature selection in enhancing clustering performance. The choice of graph-based learning methods, such as ECGFS and SOGFS, demonstrates the advantages of our approach in preserving local structure. Subspace-based learning methods, such as VCSDFS and RSPCA, help validate our method’s effectiveness in dimensionality reduction. Additionally, feature selection methods, including MCFS, UDFS, UFS^2^, and UDS^2^FS, allow us to verify that the feature subset we select is superior.

Baseline: The k-means clustering technique is directly applied to the original data clusters.

MCFS [15]: A two-step feature selection framework is accomplished by integrating spectral analysis and sparse learning.

UDFS [44]: A local discriminative UFS model emphasizes discriminative information and feature correlations to identify the most distinguishing features.

SOGFS [45]: This model can adaptively preserve local structure by constructing a more accurate similarity matrix to emphasize more distinguishing features.

EGCFS [46]: Adaptive graph learning is constructed to select distinguishing features.

VCSDFS [47]: A variance–covariance is established to redefine the subspace distances so as to eliminate the irrelevant features.

UFS^2^ [48]: This uses binary vectors in k-means to select more accurate numbers of features for clustering.

UDS^2^FS [49]: Training soft labels is designed to guide the feature selection process to identify more discriminative subspaces.

RSPCA [50]: The σ-norm was employed as the reconstruction error, while the ℓ_2,0_-norm constraint was applied to the subspace projection in the feature selection task.

### 4.3. Experimental Settings

Regarding the parameter settings, the maximum number of iterations *NIter* is set to 30. The neighborhood size *k* for DHBWSL and all comparative methods based on graph learning is set to 5. According to the literature requirements, for methods including MCFS, UDFS, SOGFS, EGCFS, VCSDFS, UFS^2^, UDS^2^FS and RSPCA, a grid search strategy is employed to select appropriate values from the set {10^−4^, 10^−3^, 10^−2^, 10^−1^, 10^0^, 10^1^, 10^2^, 10^3^, 10^4^} for parameters that need adjustment. For DHBWSL, there are four regularization parameters: data graph regularization parameter *β*, feature graph regularization parameter *γ*, sparsity parameter *λ*, and orthogonal parameter *δ*, which is set to 1. To ensure fairness in comparative experiments, a parameter search is conducted for *β*, *γ*, and *λ* within the range {10^−4^, 10^−3^, 10^−2^, 10^−1^, 10^0^, 10^1^, 10^2^, 10^3^, 10^4^}. The selected range of values for the number of features *l* is {20, 30, 40, 50, 60, 70, 80, 90, 100}. Due to the dependency of k-means clustering results on initialization, we computed the average of 20 runs to obtain the final results for ACC and NMI.

### 4.4. Clustering Results and Analysis

**Figure 2:**
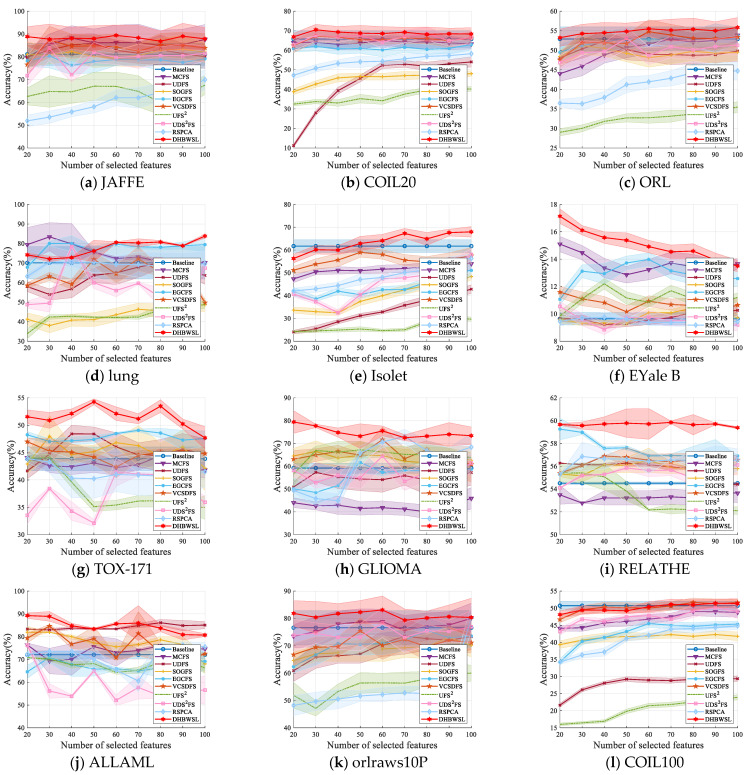
The ACC of all the algorithms for selecting different numbers of features on the 12 datasets.

**Figure 3:**
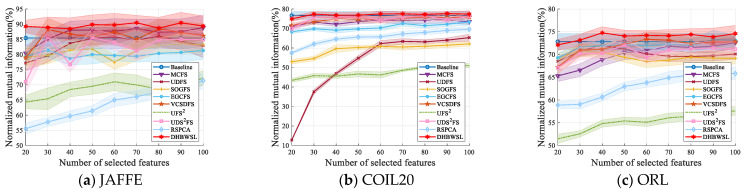

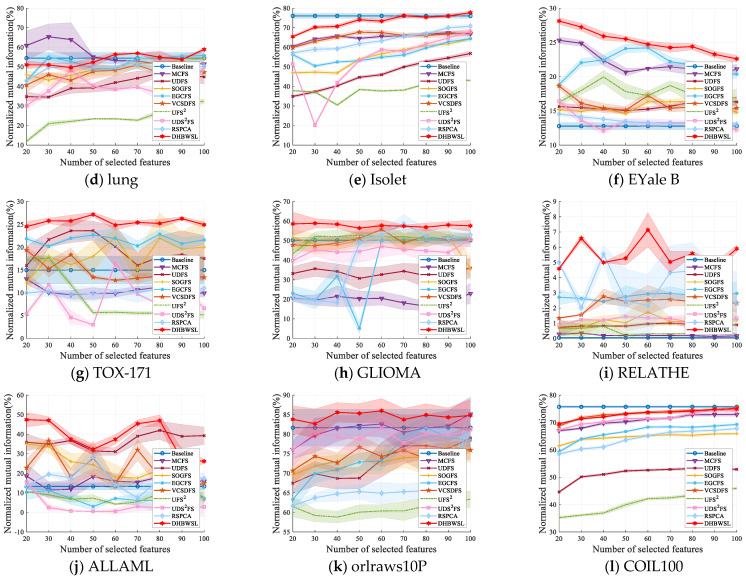
The NMI of all the algorithms for selecting different numbers of features on the 12 datasets.

The comparative clustering experiments are conducted in terms of ACC and NMI on 12 distinct datasets, as illustrated in Table 4 and Table 5. Due to the sensitivity of k-means clustering to initialization, we compute the average and standard deviation of ACC and NMI of twenty independent results, as detailed in Table 4 and Table 5. For clarity in comparing results, we highlight the best ACC and NMI values in bold and underline the second-best values. Furthermore, Figure 2 and Figure 3 analyze the variation in ACC and NMI with changes in the number of features. In Figure 2 and Figure 3, the x-coordinate represents the number of selected features, while the y-coordinate represents the value of ACC or NMI. From the experimental results presented in Table 4 and Table 5 and Figure 2 and Figure 3, it is evident that in most cases, DHBWSL achieves higher ACC and NMI values compared to other compared algorithms. This significantly exhibits its effectiveness in selecting features and its exceptional capabilities in graph learning. Detailed conclusions are provided below:

(1) Overall, most UFS methods outperform Baseline on the majority of datasets. This performance difference highlights the ability of these UFS methods to enhance clustering performance by eliminating noise and redundant features.

(2) The results presented in Table 4 and Table 5 demonstrate that our proposed DHBWSL achieves significant performance improvements compared to other comparative methods, effectively validating the superiority of DHBWSL. Specifically, compared to Baseline, MCFS, UDFS, SOGFS, EGCFS, VCSDFS, UFS^2^, UDS^2^FS and RSPCA, DHBWSL shows substantial increases in ACC by 20.20%, 33.50%, 25.14%, 35.15%, 17.70%, 11.65%, 37.53%, 15.00% and 19.46%, respectively. This indicates that DHBWSL better preserves local structural information in both data and feature spaces while effectively eliminating noise and redundancy in unlabeled data.

(3) In various real datasets encompassing diverse scenarios such as images, text, and videos, DHBWSL consistently outperforms other comparative approaches, effectively validating its superiority. Particularly on the Isolet dataset, while other methods fail to reach Baseline levels in terms of ACC and NMI, DHBWSL surpasses the Baseline performance. This outcome convincingly demonstrates the effectiveness of DHBWSL. The success is attributed to learning a more stable dual high-order graph structure and a well-sparse feature selection matrix, and thus the selected features are of higher quality, leading to more stable results in k-means clustering.

(4) The findings presented in Table 6 indicate that DHBWSL is notably competitive in terms of computational efficiency when juxtaposed with the majority of the algorithms evaluated. In particular, there is a marked decrease in time cost when compared to earlier adaptive graph learning approaches like SOGFS and EGCFS. While DHBWSL might incur a marginally higher time cost than certain subspace learning techniques such as VCSDFS, UDS^2^FS, and RSPCA, it nonetheless maintains superior computational efficiency and attains the highest level of clustering accuracy. This advantage is particularly pronounced when DHBWSL is contrasted with conventional methods, including Baseline, MCFS, and UDFS.

(5) Specifically, even compared to the latest UFS methods like EGCFS, VCSDFS, UFS^2^, UDS^2^FS and RSPCA, DHBWSL demonstrates superior clustering performance. This excellent performance is attributed to the high-order adjacency learning that facilitates learning a more stable high-order graph structure for guiding feature selection for unlabeled data compared to other comparative subspace learning methods such as VCSDFS, UDS^2^FS and RSPCA.

(6) DHBWSL performs worse than VCSDFS on the COIL100 dataset, likely because VCSDFS leverages the intrinsic statistical information and feature correlation more effectively through the Variance–Covariance Subspace Distance framework. This allows for better identification of the representative feature subset with minimum norm properties during feature selection, leading to improved dimensionality reduction and subspace learning performance. By contrast, DHBWSL is not confined to relying exclusively on statistical information. Instead, it initiates from the geometric structure of the data and, through the construction of a double high-order graph, more comprehensively captures the higher-order local structure features.

(7) To elucidate the marked enhancement in the clustering outcomes of DHBWSL as depicted in Table 7 and Table 8, a statistical analysis was conducted comparing the results of DHBWSL against those of the comparative algorithms. Specifically, a paired *t*-test was employed. Each algorithm was required to independently perform clustering 20 times to obtain the average results presented in Table 4 and Table 5, with these 20 results serving as the basis for the paired *t*-test. Observing the h and *p* values obtained from the statistical experiments, an h value of 0 indicates that the null hypothesis cannot be rejected at a significance level of 5%. Conversely, an h value of 1 indicates that the null hypothesis can be rejected at the 5% level. The *p*-value represents the significance level. When *h* = *1* and the *p*-value is small, it is generally concluded that there is a difference between the two samples, suggesting a notable improvement in the results of DHBWSL. Table 7 and Table 8 present the paired *t*-tests for DHBWSL and the comparative algorithms across all datasets. Table 7 and Table 8 demonstrate that in the paired *t*-tests, *h* = *1* and the *p*-values are small for the majority of datasets. These results indicate that, compared to other algorithms, there is a significant difference between the ACC and NMI of DHBWSL and the comparative algorithms, illustrating a substantial improvement in DHBWSL and validating its superiority.

**Table 4:** The clustering performance of compared methods on ACC ± STD% on 12 datasets.

Datasets	Baseline	MCFS	UDFS	SOGFS	EGCFS	VCSDFS	UFS^2^	UDS^2^FS	RSPCA	DHBWSL
JAFFE	81.10 ±4.85 (all)	88.24 ±5.40 (40)	85.77 ±4.31 (50)	83.29 ±6.15 (90)	80.40 ±5.29 (30)	87.37 ±5.97 (30)	67.58 ±4.06 (100)	83.76 ±5.40 (30)	69.95 ±4.23 (100)	**89.41** **±4.54** **(60)**
COIL20	65.75 ±4.16 (all)	65.14 ±2.53 (60)	54.05 ±2.63 (100)	48.04 ±1.40 (100)	62.98 ±2.87 (100)	67.16 ±2.81 (30)	40.25 ±1.18 (90)	68.43 ±3.36 (80)	58.26 ±1.85 (100)	**70.52** **±2.71** **(30)**
ORL	52.90 ±3.08 (all)	53.86 ±1.65 (100)	52.39 ±2.48 (40)	51.20 ±2.21 (30)	53.73 ±2.18 (100)	54.77 ±3.03 (60)	35.49 ±1.35 (100)	53.09 ±2.93 (50)	44.73 ±2.05 (90)	**55.85** **±2.53** **(100)**
lung	70.10 ±8.22 (all)	83.42 ±7.23 (30)	70.15 ±2.82 (80)	48.67 ±3.42 (100)	80.02 ±1.26 (30)	72.17 ±6.20 (70)	48.65 ±1.87 (100)	78.47 ±0.70 (40)	70.62 ±1.34 (30)	**83.82** **±1.03** **(100)**
Isolet	61.73 ±2.77 (all)	54.16 ±2.53 (90)	42.83 ±1.89 (100)	48.35 ±1.51 (100)	51.07 ±2.93 (100)	58.98 ±2.11 (50)	30.44 ±1.27 (90)	58.01 ±2.45 (100)	56.28 ±1.89 (100)	**67.97** **±2.29** **(100)**
EYale B	9.64 ±0.45 (all)	15.10 ±0.56 (20)	10.26 ±0.39 (100)	10.42 ±0.35 (80)	13.98 ±0.48 (60)	11.59 ±0.41 (20)	12.20 ±0.38 (40)	10.54 ±0.35 (20)	9.76 ±0.33 (40)	**17.14** **±0.53** **(20)**
TOX_171	43.86 ±2.17 (all)	44.42 ±1.27 (80)	48.42 ±1.59 (40)	47.98 ±3.80 (70)	49.06 ±0.26 (70)	46.99 ±0.46 (20)	44.06 ±0.58 (30)	41.43 ±2.57 (60)	43.92 ±0.73 (20)	**54.27** **±0.52** **(50)**
GLIOMA	59.20 ±2.19 (all)	45.90 ±4.70 (100)	57.30 ±4.17 (30)	66.50 ±4.58 (30)	61.70 ±4.46 (50)	71.70 ±5.44 (60)	67.20 ±3.75 (50)	64.40 ±5.05 (60)	73.10 ±3.08 (70)	**79.40** **±4.95** **(20)**
RELATHE	54.51 ±0.10 (all)	53.72 ±0.76 (90)	56.27 ±0.21 (50)	56.53 ±1.07 (60)	59.26 ±0.95 (20)	56.90 ±0.03 (40)	55.41 ±0.88 (30)	56.11 ±0.81 (100)	56.85 ±1.24 (30)	**59.85** **±0.12** **(70)**
ALLAML	72.08 ±1.62 (all)	76.39 ±0.78 (20)	86.11 ±1.42 (80)	81.94 ±2.60 (60)	70.90 ±2.27 (30)	84.65 ±0.71 (30)	70.83 ±0.01 (20)	76.25 ±0.62 (20)	76.32 ±1.39 (90)	**89.24** **±1.18** **(20)**
orlraws10P	76.60 ±6.17 (all)	80.05 ±5.49 (100)	73.55 ±6.60 (70)	73.00 ±3.93 (90)	75.60 ±5.08 (80)	75.45 ±4.68 (50)	60.00 ±2.87 (100)	78.35 ±5.32 (60)	80.20 ±4.81 (100)	**82.30** **±4.23** **(50)**
COIL100	50.72 ±1.30 (all)	49.01 ±1.21 (80)	29.39 ±0.61 (90)	42.30 ±1.09 (90)	45.49 ±1.12 (60)	**51.69** **±1.27** **(80)**	23.93 ±0.74 (100)	50.15 ±1.32 (90)	44.80 ±0.93 (100)	51.47 ±1.18 (90)

**Table 5:** The clustering performance of compared methods on NMI ± STD% on 12 datasets.

Datasets	Baseline	MCFS	UDFS	SOGFS	EGCFS	VCSDFS	UFS^2^	UDS^2^FS	RSPCA	DHBWSL
JAFFE	85.43 ±3.51 (all)	88.88 ±4.03 (100)	86.06 ±3.51 (80)	83.58 ±3.26 (90)	81.48 ±2.59 (30)	88.44 ±3.20 (80)	72.25 ±2.38 (100)	85.79 ±3.36 (30)	71.38 ±2.56 (100)	**90.48** **±2.40** **(70)**
COIL20	76.69 ±1.99 (all)	74.42 ±1.50 (60)	65.33 ±1.75 (100)	62.12 ±1.20 (100)	73.36 ±1.61 (100)	75.84 ±1.31 (80)	51.27 ±0.81 (90)	76.60 ±1.75 (80)	69.56 ±1.35 (100)	**77.67** **±1.09** **(90)**
ORL	72.83 ±1.74 (all)	72.61 ±1.13 (100)	72.09 ±1.39 (40)	70.88 ±1.37 (30)	73.13 ±1.17 (100)	73.34 ±1.63 (60)	57.60 ±0.93 (100)	72.06 ±1.56 (50)	66.07 ±1.50 (90)	**74.77** **±1.31** **(40)**
lung	54.47 ±2.84 (all)	**65.38** **±6.55** **(30)**	46.72 ±1.36 (80)	54.21 ±2.54 (100)	55.80 ±2.69 (100)	50.91 ±1.58 (70)	32.46 ±1.26 (100)	50.06 ±1.47 (100)	50.97 ±2.24 (70)	58.91 ±0.89 (100)
Isolet	76.06 ±1.26 (all)	67.43 ±0.92 (90)	56.84 ±1.27 (100)	64.37 ±0.71 (100)	64.43 ±1.30 (100)	67.74 ±1.09 (50)	43.56 ±0.65 (90)	68.11 ±1.46 (100)	70.81 ±0.97 (100)	**77.75** **±0.76** **(100)**
EYale B	12.77 ±0.52 (all)	25.31 ±0.48 (20)	16.46 ±0.47 (90)	16.41 ±0.56 (80)	24.20 ±0.60 (60)	18.64 ±0.41 (20)	19.95 ±0.95 (40)	16.42 ±0.50 (20)	14.54 ±0.24 (20)	**28.13** **±0.48** **(20)**
TOX_171	14.98 ±3.03 (all)	13.03 ±1.19 (20)	23.61 ±2.09 (50)	22.20 ±1.74 (60)	22.85 ±1.12 (80)	19.40 ±0.66 (20)	17.66 ±0.80 (30)	16.54 ±0.82 (60)	11.00 ±1.60 (100)	**27.16** **±0.46** **(50)**
GLIOMA	50.20 ±1.60 (all)	22.94 ±5.08 (100)	36.05 ±4.99 (100)	52.37 ±3.62 (90)	51.23 ±1.53 (90)	55.72 ±3.37 (60)	52.75 ±1.91 (50)	50.68 ±2.39 (100)	58.60 ±4.82 (70)	**58.89** **±2.40** **(30)**
RELATHE	0.05 ±0.02 (all)	0.35 ±0.01 (30)	1.01 ±0.09 (70)	1.73 ±0.95 (60)	2.96 ±0.75 (100)	2.76 ±0.49 (40)	0.79 ±0.41 (40)	1.43 ±0.09 (50)	5.54 ±0.57 (40)	**7.14** **±1.12** **(60)**
ALLAML	13.33 ±1.84 (all)	18.63 ±6.11 (30)	41.97 ±4.63 (80)	34.99 ±1.24 (20)	11.22 ±2.39 (100)	36.68 ±2.05 (30)	10.94 ±2.20 (90)	15.58 ±0.62 (20)	28.40 ±5.96 (50)	**47.42** **±3.72** **(20)**
orlraws10P	81.76 ±4.70 (all)	85.17 ±4.34 (100)	79.05 ±3.36 (100)	77.19 ±2.37 (90)	81.69 ±3.14 (80)	76.69 ±3.04 (50)	63.29 ±2.13 (100)	81.59 ±3.00 (60)	84.77 ±3.04 (100)	**85.34** **±2.48** **(50)**
COIL100	**75.73** **±0.34** **(all)**	72.88 ±0.6 (100)	53.15 ±0.53 (80)	65.91 ±0.37 (100)	69.29 ±0.36 (100)	74.51 ±0.54 (80)	45.97 ±0.46 (100)	74.42 ±0.50 (90)	67.78 ±0.40 (100)	74.95 ±6.30 (100)

**Table 6:** Computation time (seconds) of different methods on real-world datasets.

Datasets	Baseline	MCFS	UDFS	SOGFS	EGCFS	VCSDFS	UFS^2^	UDS^2^FS	RSPCA	DHBWSL
JAFFE	0.06	0.86	1.48	4.40	1.12	0.38	1.59	1.52	0.73	1.32
COIL20	0.47	6.64	13.94	96.53	19.71	1.28	14.82	5.85	1.38	10.91
ORL	0.18	2.03	2.79	7.96	3.51	1.24	3.37	3.13	1.19	4.18
lung	2.75	6.04	68.34	2408.09	93.14	8.96	5.24	41.52	27.92	30.28
Isolet	0.46	7.07	13.41	23.73	18.86	1.40	11.24	1.68	0.77	8.89
EYale B	1.45	3.26	24.92	65.17	58.57	1.43	46.04	2.59	1.68	34.10
TOX_171	25.18	1.28	873.81	2834.97	1242.36	27.17	17.83	180.34	135.95	405.69
GLIOMA	12.39	0.89	607.04	2314.70	597.92	16.81	3.72	87.42	36.44	245.35
RELATHE	11.42	11.86	418.58	944.73	579.20	16.57	37.48	143.23	62.56	338.26
ALLAML	47.20	6.75	1298.96	10,709.38	1145.44	132.98	7.40	453.89	149.60	243.76
orlraws10P	163.67	15.68	2566.06	19,028.84	4159.70	87.63	22.62	805.71	451.26	1746.03
COIL100	45.42	31.35	320.69	962.12	2015.05	1.33	88.20	12.67	11.09	311.45

**Table 7:** The paired *t*-test result of ACC of DHBWSL and comparison algorithms on all datasets.

Datasets		Baseline	MCFS	UDFS	SOGFS	EGCFS	VCSDFS	UFS^2^	UDS^2^FS	RSPCA
JAFFE	p h	0.0033 1	4.9999 × 10^−9^ 1	1.1243 × 10^−11^ 1	7.6029 × 10^−16^ 1	0.0088 1	8.1090 × 10^−4^ 1	1.7584 × 10^−18^ 1	6.6927 × 10^−5^ 1	2.7192 × 10^−10^ 1
COIL20	p h	0.0083 1	2.2206 × 10^−7^ 1	2.7762 × 10^−29^ 1	2.1609 × 10^−44^ 1	3.2358 × 10^−35^ 1	1.8528 × 10^−6^ 1	7.8998 × 10^−41^ 1	0.0109 1	2.0050 × 10^−17^ 1
ORL	p h	4.1188 × 10^−4^ 1	6.1222 × 10^−7^ 1	1.6053 × 10^−11^ 1	9.9364 × 10^−5^ 1	0.0046 1	0.2030 0	5.2170 × 10^−28^ 1	2.5978 × 10^−5^ 1	2.4171 × 10^−16^ 1
lung	p h	1.7868 × 10^−7^ 1	1.4484 × 10^−16^ 1	2.9665 × 10^−8^ 1	1.7777 × 10^−12^ 1	9.0574 × 10^−23^ 1	0.0356 1	3.4934 × 10^−19^ 1	1.8391 × 10^−6^ 1	5.7823 × 10^−10^ 1
Isolet	p h	5.9167 × 10^−28^ 1	2.1798 × 10^−14^ 1	2.7699 × 10^−25^ 1	1.9143 × 10^−36^ 1	1.4784 × 10^−35^ 1	1.4844 × 10^−11^ 1	5.9769 × 10^−40^ 1	2.9701 × 10^−7^ 1	7.7368 × 10^−19^ 1
EYale B	p h	1.6388 × 10^−29^ 1	1.0569 × 10^−8^ 1	1.4683 × 10^−28^ 1	2.1944 × 10^−41^ 1	2.6630 × 10^−32^ 1	9.4853 × 10^−31^ 1	1.6083 × 10^−26^ 1	4.4488 × 10^−24^ 1	4.5104 × 10^−35^ 1
TOX_171	p h	3.8869 × 10^−6^ 1	0.0093 1	1.7762 × 10^−6^ 1	4.5149 × 10^−16^ 1	5.0969 × 10^−11^ 1	4.9939 × 10^−9^ 1	6.9265 × 10^−13^ 1	5.2356 × 10^−9^ 1	0.0219 1
GLIOMA	p h	4.3778 × 10^−13^ 1	7.9113 × 10^−26^ 1	7.3934 × 10^−22^ 1	7.5027 × 10^−14^ 1	2.8855 × 10^−23^ 1	9.1073 × 10^−17^ 1	2.4122 × 10^−16^ 1	6.6404 × 10^−8^ 1	7.1229 × 10^−18^ 1
RELATHE	p h	0.0371 1	5.2259 × 10^−45^ 1	1.9256 × 10^−81^ 1	4.8465 × 10^−65^ 1	1.2509 × 10^−74^ 1	6.0813 × 10^−7^ 1	3.2163 × 10^−65^ 1	5.9863 × 10^−10^ 1	0.0069 1
ALLAML	p h	2.0298 × 10^−37^ 1	6.0648 × 10^−20^ 1	2.9455 × 10^−51^ 1	1.3964 × 10^−49^ 1	1.1054 × 10^−9^ 1	5.1027 × 10^−16^ 1	5.5017 × 10^−34^ 1	5.9833 × 10^−64^ 1	2.2404 × 10^−30^ 1
orlraws10P	p h	5.5865 × 10^−18^ 1	1.5253 × 10^−21^ 1	5.3369 × 10^−13^ 1	1.6683 × 10^−17^ 1	1.3257 × 10^−15^ 1	1.1109 × 10^−11^ 1	2.3574 × 10^−15^ 1	1.2112 × 10^−19^ 1	9.2441 × 10^−18^ 1
COIL100	p h	1.9101 × 10^−21^ 1	1.0699 × 10^−15^ 1	1.1474 × 10^−45^ 1	3.6026 × 10^−13^ 1	5.8528 × 10^−26^ 1	3.1366 × 10^−23^ 1	4.8302 × 10^−49^ 1	1.1606 × 10^−14^ 1	3.6670 × 10^−32^ 1

**Table 8:** The paired *t*-test result of NMI of DHBWSL and comparison algorithms on all datasets.

Datasets		Baseline	MCFS	UDFS	SOGFS	EGCFS	VCSDFS	UFS^2^	UDS^2^FS	RSPCA
JAFFE	p h	5.0954 × 10^−9^ 1	4.5287 × 10^−14^ 1	1.2985 × 10^−18^ 1	2.1976 × 10^−25^ 1	4.3009 × 10^−7^ 1	1.7813 × 10^−5^ 1	4.0952 × 10^−18^ 1	5.8906 × 10^−10^ 1	3.4081 × 10^−16^ 1
COIL20	p h	0.9210 0	1.8565 × 10^−11^ 1	8.3184 × 10^−35^ 1	7.7725 × 10^−54^ 1	1.3827 × 10^−42^ 1	1.5602 × 10^−12^ 1	2.1203 × 10^−48^ 1	0.1891 0	1.5006 × 10^−26^ 1
ORL	p h	1.3472 × 10^−6^ 1	3.8923 × 10^−6^ 1	4.2445 × 10^−17^ 1	7.9821 × 10^−9^ 1	1.1772 × 10^−5^ 1	0.1182 0	1.1551 × 10^−37^ 1	6.1003 × 10^−6^ 1	9.6748 × 10^−23^ 1
lung	p h	2.4998 × 10^−18^ 1	1.4410 × 10^−20^ 1	2.0524 × 10^−8^ 1	2.4254 × 10^−14^ 1	2.1454 × 10^−24^ 1	3.2489 × 10^−7^ 1	1.5531 × 10^−20^ 1	3.3657 × 10^−6^ 1	6.0400 × 10^−16^ 1
Isolet	p h	6.9752 × 10^−38^ 1	5.3449 × 10^−22^ 1	3.7346 × 10^−36^ 1	5.3022 × 10^−43^ 1	8.5538 × 10^−47^ 1	7.4706 × 10^−8^ 1	2.4377 × 10^−53^ 1	0.0243 1	7.7473 × 10^−30^ 1
EYale B	p h	7.9018 × 10^−44^ 1	7.7974 × 10^−7^ 1	4.9514 × 10^−35^ 1	4.1198 × 10^−45^ 1	6.5993 × 10^−40^ 1	7.0041 × 10^−42^ 1	2.5865 × 10^−28^ 1	3.1037 × 10^−37^ 1	1.0691 × 10^−47^ 1
TOX_171	p h	1.2300 × 10^−15^ 1	1.0453 × 10^−4^ 1	0.0275 1	1.8397 × 10^−22^ 1	1.1552 × 10^−26^ 1	9.4111 × 10^−18^ 1	6.4780 × 10^−21^ 1	4.0051 × 10^−12^ 1	0.0073 1
GLIOMA	p h	1.5787 × 10^−4^ 1	2.1801 × 10^−31^ 1	1.1535 × 10^−32^ 1	3.4468 × 10^−14^ 1	7.5197 × 10^−34^ 1	4.1431 × 10^−7^ 1	1.7450 × 10^−20^ 1	0.0239 1	5.2694 × 10^−6^ 1
RELATHE	p h	0.0153 1	1.1618 × 10^−74^ 1	1.0218 × 10^−93^ 1	1.2836 × 10^−55^ 1	5.8358 × 10^−90^ 1	2.2538 × 10^−7^ 1	3.5715 × 10^−53^ 1	6.3098 × 10^−8^ 1	5.7191 × 10^−13^ 1
ALLAML	p h	2.4430 × 10^−31^ 1	5.4551 × 10^−19^ 1	4.3531 × 10^−39^ 1	1.8018 × 10^−62^ 1	5.2290 × 10^−5^ 1	1.7163 × 10^−11^ 1	1.3913 × 10^−24^ 1	8.8682 × 10^−95^ 1	1.7695 × 10^−25^ 1
orlraws10P	p h	2.8720 × 10^−22^ 1	2.2141 × 10^−25^ 1	2.8931 × 10^−17^ 1	2.6862 × 10^−18^ 1	2.1888 × 10^−17^ 1	3.8995 × 10^−14^ 1	5.8639 × 10^−6^ 1	1.4837 × 10^−23^ 1	9.1751 × 10^−21^ 1
COIL100	p h	2.1942 × 10^−40^ 1	7.4485 × 10^−31^ 1	1.7060 × 10^−56^ 1	3.3267 × 10^−28^ 1	2.7279 × 10^−38^ 1	5.0523 × 10^−33^ 1	1.9495 × 10^−60^ 1	2.0572 × 10^−33^ 1	9.8478 × 10^−42^ 1

### 4.5. Visualization on Fashion MNIST

The Fashion MNIST dataset encompasses 70,000 grayscale images, distributed across 10 distinct categories. Specifically, it consists of a training set with 60,000 samples and a test set comprising 10,000 samples, where each sample is represented as a 28 × 28 grayscale image. In the present experiment, we select images from the test set to utilize as training samples for the purpose of conducting tests. In the following experiments, we verify the interpretability of the feature selection task in the Fashion MNIST dataset. We visualize the feature subsets derived from the feature selection methods on the Fashion MNIST dataset using t-SNE. In our experiments, we compare the Baseline, UDS^2^FS, and DHBWSL methods. For the Baseline method, the entire set of features is used as the feature subset to represent the original dataset, whereas, for both UDS^2^FS and DHBWSL, we select the top 100 features as the feature subsets.

**Figure 4:**
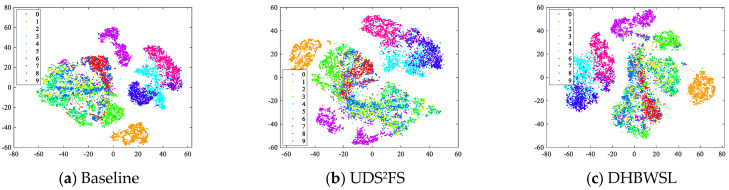
The 2D demonstration of the Fashion MNIST dataset.

As the experimental results show in Figure 4a,b, the intraclass distance is very small, indicating that neither Baseline nor UDS^2^FS methods can effectively distinguish between different classes. In contrast, Figure 4c demonstrates that our DHBWSL successfully reduces the intra-class distance while enhancing the inter-class distance. Especially when the coordinate scale is the same as that in Figure 4a, the overall spatial structure of DHBWSL remains consistent compared to Baseline. This further confirms that DHBWSL effectively considers higher-order relationships among data points, thereby more accurately preserving the local structure of the original data. Concurrently, it minimizes class clustering, maximizes inter-class distances, and maintains the spatial geometry of the data, while selecting discriminative features.

### 4.6. Two-Moon Dataset and Noise Test

**Figure 5:**
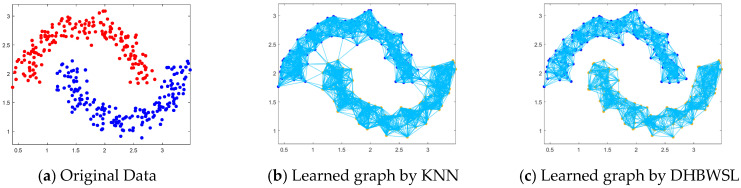
Learned graphs on the Two-moon synthetic data.

In this segment, we assess the local learning ability of DHBWSL using a synthetic dataset, specifically the Two-moon dataset. To ensure a fair evaluation, the neighbor count is fixed at 20. As depicted in Figure 5a, the original Two-moon dataset features two distinct classes, represented by red and blue data points, each comprising 90 samples, with a noise level of 0.12% incorporated. Figure 5b presents the visualization of the similarity graph constructed via the K-nearest neighbor (KNN) approach. Notably, there are instances where multiple lines link data points from separate categories. This implies that certain data points have nearest neighbors belonging to different classes, underscoring the adverse impact of noise features on the sample’s similarity structure. Such noise compromises the chart’s reliability. Conversely, Figure 5c reveals a starkly clear demarcation between the two distinct categories, the red and blue data points. This clarity indicates that DHBWSL’s clustering performance is nearly on par with that of the pristine Two-moon dataset. The rationale behind this outcome is DHBWSL’s capability to dynamically learn a high-quality similarity graph. This ensures that the resultant graph is exclusively composed of the two categories, effectively eradicating any connecting lines between the red and blue data points.

**Figure 6:**
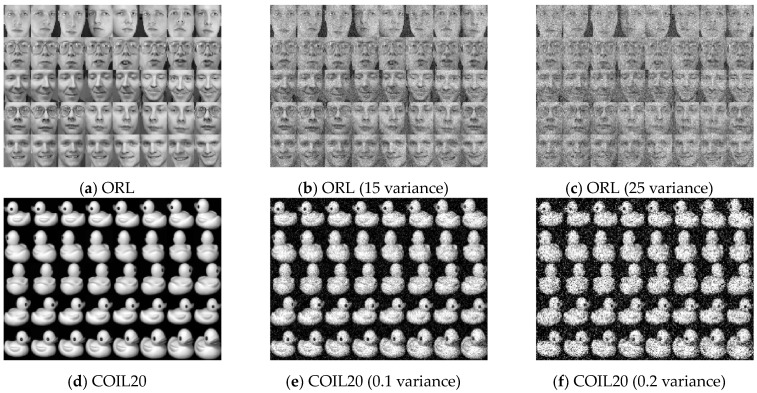
Samples from ORL and COIL20 datasets with Gaussian noise with different variances.

We conduct noise tests to further confirm the effectiveness of DHBWSL, with four noisy datasets generated by adding Gaussian noise with variances of 15 and 25 to ORL and variances of 0.1 and 0.2 to the COIL20 dataset. These noises are randomly added to the original image as shown in Figure 6b,c,e,f. In Figure 6c,f, significant blurring effects on facial and image features can be observed. As the clustering results depicted in Table 9 show, it is evident that under various noise conditions, DHBWSL consistently exhibits superiority to other compared methods. This further validates the enhanced robustness of DHBWSL in its ability to learn a more stable dual high-order graph structure.

### 4.7. Ablation Study

**Figure 7:**
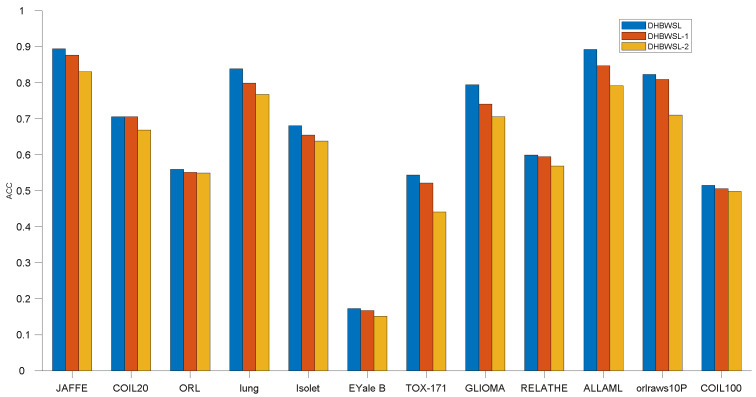
Ablation experiment on DHBWSL and its variants in terms of ACC.

**Figure 8:**
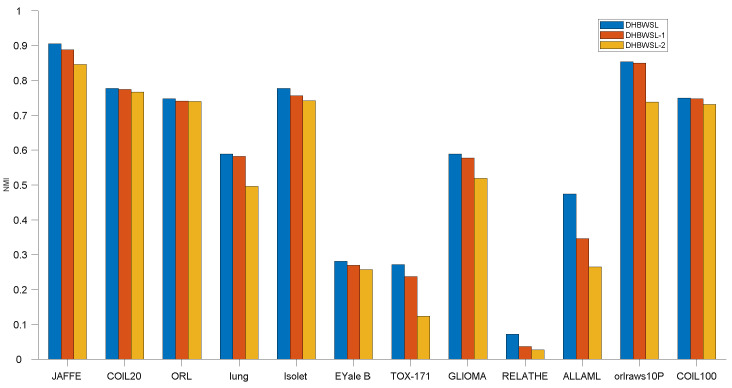
Ablation experiment on DHBWSL and its variants in terms of NMI.

In this section, we conducted ablation experiments to validate the effectiveness of dual high-order graph regularization and the *ℓ*_2,1_-norm constraint. We considered two specific scenarios of the DHBWSL, denoted as DHBWSL-1 and DHBWSL-2. For DHBWSL-1, we set the *ℓ*_2,1_-norm parameter *λ* = 0 in Equation (14). For DHBWSL-2, we set the dual high-order graph regularization parameters *γ* = 0 and *β* = 0 in Equation (14). The results are shown in Figure 7 and Figure 8. It can be observed from the figures that DHBWSL achieves higher clustering accuracy across all datasets compared to DHBWSL-1 and DHBWSL-2. Regarding clustering performance, DHBWSL-1 performs similarly to DHBWSL, while DHBWSL-2 exhibits the lowest performance. Therefore, we conclude that in our approach, dual high-order graph regularization has more influence than sparse *ℓ*_2,1_-norm constraint on the clustering performance, which is attributed to the fact that dual high-order graph regularization preserves the local geometric structure in the data space and feature space.

### 4.8. Parameters Sensitivity Analysis

There are four parameters in DHBWSL, i.e., the orthogonality constraint parameter *δ* set to 1 and three adjusted regularization parameters: the dual-graph regularization parameters *γ* and *β*, and the sparse constraint parameter *λ*. From the ablation analysis in Section 4.6, the dual-graph regularization term significantly contributes to clustering performance. Therefore, we primarily conducted sensitivity experiments on the parameters *γ* and *β*. For convenience, we fixed the number of selected features at 100 and employed a grid search to adjust the values of *γ* and *β* within the range {10^−4^, 10^−3^, 10^−2^, 10^−1^, 10^0^, 10^1^, 10^2^, 10^3^, 10^4^}, while keeping *λ* = 1 and *δ* = 1 fixed.

**Figure 9:**
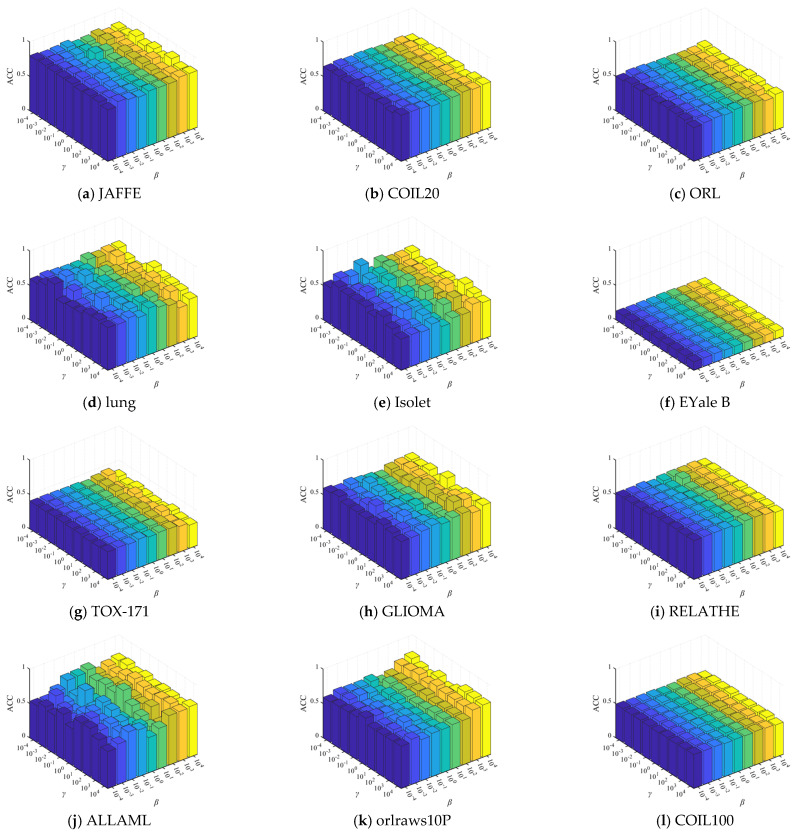
The ACC of DHBWSL on 12 datasets under values of *γ* and *β*(*λ* = 1, *δ* = 1).

**Figure 10:**
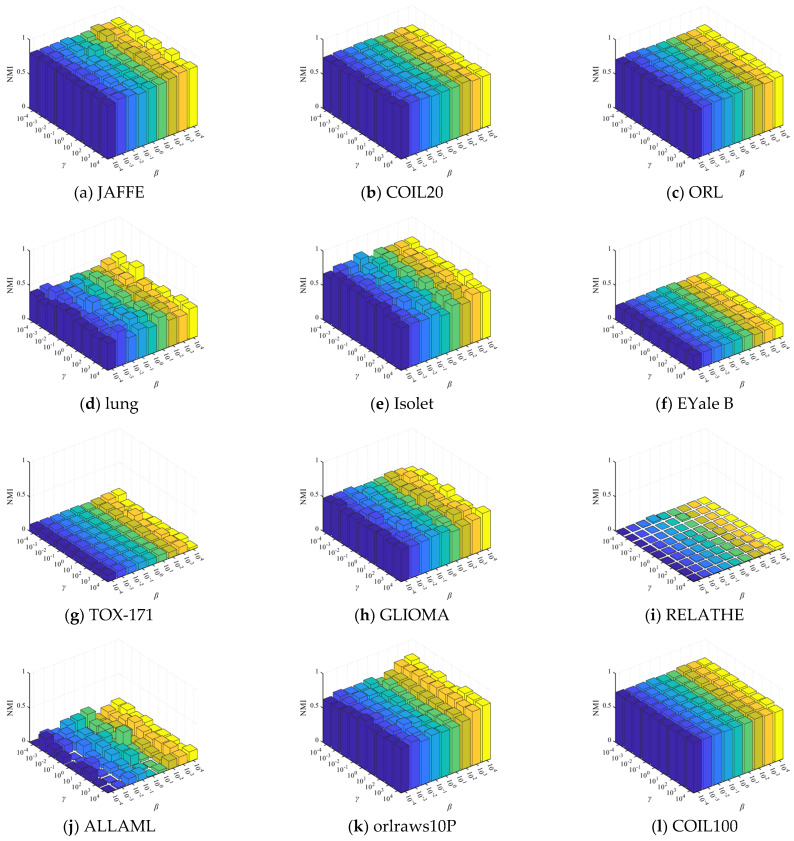
The NMI of DHBWSL on 12 datasets under values of *γ* and *β*(*λ* = 1, *δ* = 1).

From Figure 9 and Figure 10, it can be observed that there is no significant overall variation in the ACC and NMI values across the 12 datasets with changes in parameters. This implies that DHBWSL is not highly sensitive to parameter variations to a certain extent, that is, it maintains relative stability over a wide range of parameter values. Hence, the appropriate ranges for the parameters *γ* and *β* are {10^−4^, 10^−2^} and {10^2^, 10^4^}, respectively. Within these ranges, it is easier to achieve good performance, suggesting that DHBWSL can consistently deliver effective results under various parameter settings.

### 4.9. Convergence Analysis and Computational Performance

**Figure 11:**
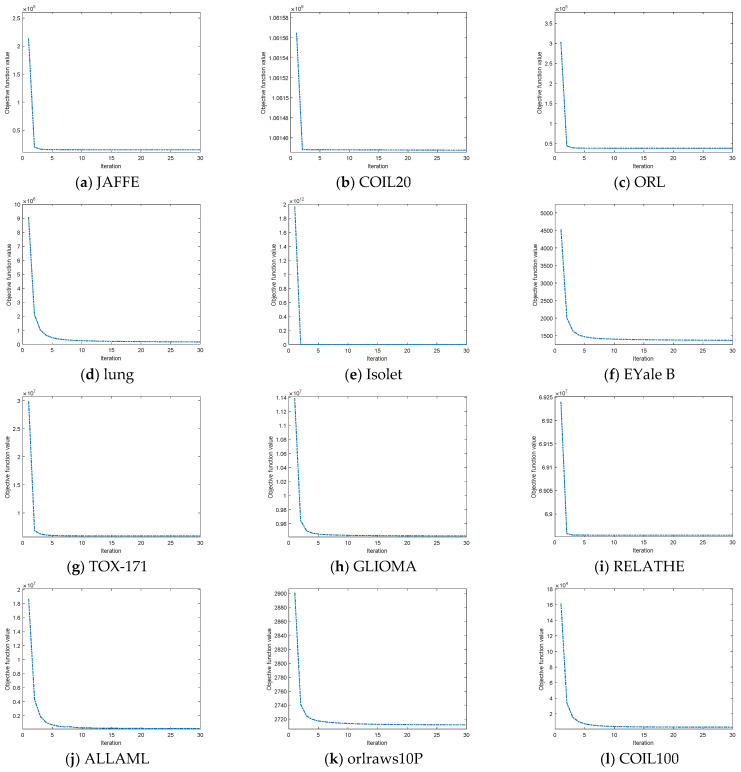
Convergence curves of the DHBWSL algorithm under different iterations on 12 different datasets.

The convergence of DHBWSL is empirically evaluated in Figure 11, in which convergence curves are illustrated on 12 public datasets with the *y*-axis indicating the objective function value. It can be observed that as the number of iterations grows, the objective function value rapidly falls and converges in less than five iterations, a fact that is guaranteed theoretically in Lemma 1.

The computational complexity of eight comparison algorithms is summarized in Table 10. Where *c* is the number of clusters, *d* is the dimensionality of the original features, *l* is the number of selected features, *n* is the total number of samples, and *t* is the number of iterations in the *k*-neighborhood. In our experiments, the computational cost of subspace dimensionality reduction is *nd*, and the computational cost of the similarity matrices *W^V^* and *W^U^* in dual space is *d*^2^ + *n*^2^ + *ld*^2^ + *d*^2^*n*. The cost of sparse learning is *dl*. Therefore, the overall computational complexity of the DHBWSL algorithm is *O*(*t*(*nd* + *d*^2^ + *n*^2^ + *ld*^2^ + *d*^2^*n* + *dl*)).

## 5. Conclusions

This paper proposes an unsupervised feature selection method, called DHBWSL. Specifically, a new graph regularization term, called dual high-order graph learning, is used to extract the geometric structure information inherent in the data and feature space. Accordingly, DHBWSL integrates the dual high-order graph learning with Boolean weights so as to improve the prospecting ability of local geometry through adaptive graph learning. Extensive numerical experiments on 12 datasets are utilized to validate the superiority of the performance of DHBWSL compared with nine state-of-the-art feature selection methods.

Although DHBWSL demonstrates better performance in the trial, there are definitely opportunities for additional development in the future. Multi-view clustering, a well-known concept in data analysis, seeks to expose consistent structural information inside datasets by utilizing many data perspectives. In future work, we plan to extend the strategies proposed in this paper to existing multi-view clustering methods to further enhance their performance. In addition, a limitation of DHBWSL is the need to tune three parameters, which can be time-consuming. Therefore, in our future work, we aim to develop a new DHBWSL mechanism that eliminates the need for parameter tuning or design a novel optimization approach that can simultaneously optimize all variables.

## Figures and Tables

**Table 1:** The notations used in this paper.

Notations	Definition
*X*	The data matrix
*H*	The feature selection matrix
*V*	The coefficient matrix
*n*	The sample number
*d*	The feature quantity
*l*	The number of selected features
*k*	The number of nearest neighbors
*c*	The number of clusters
*x_i_*	The *i*-th row vector of matrix *X*
*x_j_*	The *j*-th column vector of matrix *X*
$p_{i}^{V}$	The feature Boolean variable
*W^V^*	The feature adjacency matrix
*D^V^*	The feature graph degree matrix
*L^V^*	The feature Laplacian matrix
$p_{i}^{U}$	The data Boolean variable
*W^U^*	The data adjacency matrix
*D^U^*	The data graph degree matrix
*L^U^*	The data Laplacian matrix
**1**	A column vector with all 1 s

**Table 2:** Differences between unsupervised feature selection based on subspace learning.

Methods	High-Order Graph Learning	Minimum Redundant	Boolean Weight	Sparse Regularization	Orthogonal
MFFS [20] (2015)	×	×	×	×	√
MPMR [21] (2015)	×	√	×	×	√
SGFS [27] (2016)	×	×	×	ℓ_2,1_-norm	√
LDSSL [28] (2019)	×	×	×	ℓ_1_-norm	√
LRDAGP [33] (2020)	×	×	×	ℓ_2,1_-norm	√
DGSLFS [34] (2021)	×	×	×	ℓ_2,1_-norm	√
USFN [18] (2023)	×	×	×	×	√
MODA [35] (2023)	×	×	×	×	√
RMDDA [36] (2024)	×	×	×	F-norm	√
DSSL-MR [37] (2024)	×	×	×	ℓ_2,1_-norm	×
DHBWSL	√	×	√	ℓ_2,1_-norm	√

**Table 3:** Specific information for the 12 datasets.

Datasets	Instance	Feature	Class	Type
JAFFE	213	676	10	Face images
COIL20	1440	1024	20	Object images
ORL	400	1024	40	Face images
lung	203	3312	4	Biological
Isolet	1560	617	26	Speech Signal
EYale B	2414	1024	38	Face images
TOX_171	171	5748	4	Biological
GLIOMA	50	4434	4	Biological
RELATHE	1427	4322	2	Text
ALLAML	72	7129	2	Biological
orlraws10P	100	10,304	10	Face images
COIL100	7200	1024	100	Object images

**Table 9:** The ACC and NMI of DHBWSL on four datasets with different variance noises (ACC ± STD% and NMI ± STD%).

Noise Datasets	Accuracy (%)								
	MCFS	UDFS	SOGFS	EGCFS	VCSDFS	UFS^2^	UDS^2^FS	RSPCA	DHBWSL
ORL (15 variance)	51.94 ±2.59 (100)	45.84 ±1.78 (40)	49.89 ±2.57 (100)	53.31 ±2.45 (80)	50.99 ±3.04 (90)	33.85 ±1.18 (100)	50.66 ±2.09 (90)	43.84 ±2.62 (100)	**53.36** **±2.30** **(100)**
ORL (25 variance)	44.74 ±2.40 (100)	45.01 ±2.37 (100)	44.79 ±1.95 (100)	49.45 ±2.50 (90)	46.90 ±1.55 (90)	31.54 ±1.08 (100)	43.87 ±2.43 (90)	41.30 ±2.30 (100)	**49.14** **±2.27** **(100)**
COIL20 (0.1 variance)	64.91 ±2.67 (60)	62.22 ±2.94 (100)	61.76 ±3.13 (100)	62.10 ±2.64 (80)	67.45 ±2.24 (80)	40.18 ±1.50 (100)	67.81 ±2.42 (50)	59.37 ±2.10 (100)	**69.19** **±1.82** **(80)**
COIL20 (0.2 variance)	64.32 ±3.55 (100)	62.82 ±2.80 (90)	62.99 ±2.23 (100)	62.41 ±1.72 (80)	65.94 ±2.47 (50)	37.56 ±0.99 (100)	68.33 ±1.63 (90)	58.76 ±2.03 (100)	**69.51** **±1.78** **(90)**
**Noise datasets**	**Normalized Mutual Information (%)**								
	**MCFS**	**UDFS**	**SOGFS**	**EGCFS**	**VCSDFS**	**UFS^2^**	**UDS^2^FS**	**RSPCA**	**DHBWSL**
ORL (15 variance)	71.19 ±1.39 (100)	66.92 ±0.99 (40)	70.02 ±1.09 (100)	72.10 ±0.84 (100)	70.25 ±1.77 (80)	55.80 ±1.15 (100)	70.52 ±1.37 (90)	64.82 ±1.32 (100)	**72.14** **±1.06** **(100)**
ORL (25 variance)	64.98 ±1.61 (100)	64.44 ±0.97 (100)	63.88 ±1.17 (100)	68.46 ±0.93 (90)	66.60 ±1.15 (90)	53.21 ±1.03 (100)	64.45 ±1.42 (90)	62.18 ±1.25 (100)	**68.27** **±1.66** **(90)**
COIL20 (0.1 variance)	75.10 ±0.86 (100)	71.15 ±1.28 (100)	72.15 ±1.07 (100)	72.72 ±1.16 (70)	76.28 ±0.95 (80)	50.94 ±0.90 (100)	76.80 ±1.40 (50)	69.30 ±1.36 (100)	**77.41** **±1.17** **(100)**
COIL20 (0.2 variance)	73.97 ±1.52 (100)	70.87 ±1.27 (90)	71.57 ±1.02 (80)	71.45 ±1.11 (90)	73.76 ±1.30 (80)	46.87 ±0.77 (100)	76.10 ±0.96 (90)	68.90 ±1.18 (100)	**76.49** **±1.26** **(80)**

**Table 10:** Computational complexity analysis.

Algorithms	Computational Complexity
MCFS	*O*(*t*(*dn*^2^ + *cl*^3^ + *cnl*^2^ + *dlogd*))
UDFS	*O*(*t*(*d*^3^))
SOGFS	*O*(*t*(*d*^3^ + *n*^3^))
EGCFS	*O*(*t*(*n*^3^ + *dn* + *nl*))
VCSDFS	*O*(*t*(*d*^2^))
UFS^2^	*O*(*t*(*ncd* + *ld*))
UDS^2^FS	*O*(*t*(*d*^2^*lcn* + *dn*^2^ + *ln*^2^))
RSPCA	*O*(*t*(*nd* + max(*dlk*, *dlogd*, *llogl*, *l*^3^)))
DHBWSL	*O*(*t*(*nd* + *d*^2^ + *n*^2^ + *ld*^2^ + *d*^2^*n* + *dl*))

## Data Availability

The data and code that support the findings of this study are available from the corresponding author (J.M.) upon reasonable request.
